# 

**Published:** 1956-04

**Authors:** 


### Contents

| | | Page |
|---|---|---|
| VITAL STATISTICS OF THE SOUTH-WEST IN 1954 | *Dr. A. H. Gale* | 39 |
| A MORBID VIEW OF THE OVER-65's . . . | *Dr. G. Stewart Smith* | 44 |
| THE POSITION OF NURSING IN HOSPITALS IN BRISTOL . . . . . . . | *Mr. Stephen C. Merivale* | 55 |
| PAEDIATRIC COMMENT . . . . . . . . | *A.V.N.* | 58 |
| PERSPECTIVE IN PAEDIATRICS . . . . | *Prof. W. S. Craig* | 59 |
| THE NEW OUTLOOK IN OTO-LARYNGOLOGY | *Mr. R. Scott Stevenson* | 67 |
| MATERNAL DEATH DUE TO RHEUMATIC HEART DISEASE | *Clinical Pathological Conference* | 79 |
| CORRESPONDENCE . . . . . . . . . . . . | | 82 |
| ANNOTATION . . . . . . . . . . . . <br>BRISTOL REGISTER OF CONGENITAL HEART DISEASE | | 83 |
| NEWS FROM SOCIETIES . . . . . . . . . <br>CORNWALL CLINICAL SOCIETY<br>DEVON AND EXETER MEDICO-CHIRURGICAL SOCIETY<br>WESSEX RAHERE CLUB | | 83 |
| NOTES AND NEWS . . . . . . . . . . <br>UNIVERSITY OF BRISTOL | | 84 |
| APPOINTMENTS . . . . . . . . . . . . | | 85 |

